# CD200-positive cancer associated fibroblasts augment the sensitivity of Epidermal Growth Factor Receptor mutation-positive lung adenocarcinomas to EGFR Tyrosine kinase inhibitors

**DOI:** 10.1038/srep46662

**Published:** 2017-04-21

**Authors:** Masayuki Ishibashi, Shinya Neri, Hiroko Hashimoto, Tomoyuki Miyashita, Tatsuya Yoshida, Yuka Nakamura, Hibiki Udagawa, Keisuke Kirita, Shingo Matsumoto, Shigeki Umemura, Kiyotaka Yoh, Seiji Niho, Masahiro Tsuboi, Kenkichi Masutomi, Koichi Goto, Atsushi Ochiai, Genichiro Ishii

**Affiliations:** 1 Division of Pathology, Exploratory Oncology Research and Clinical Trial Center, National Cancer Center Hospital East, Kashiwa, Chiba, Japan; 2 Department of Thoracic Oncology, National Cancer Center Hospital East, Kashiwa, Chiba, Japan; 3 Department of Pathology, Keio University School of Medicine, Shinjuku-ku, Tokyo, Japan; 4 Division of Thoracic Surgery, National Cancer Center Hospital East, Kashiwa, Chiba, Japan; 5 Division of Cancer Stem Cell, National Cancer Center Research Institute, Chuo-ku, Tokyo, Japan

## Abstract

Cancer associated fibroblasts (CAFs) play important roles in the chemotherapeutic process, especially through influencing the resistance of tumor cells to molecular targeted therapy. Here we report the existence of a special subpopulation of patient-specific-CAFs that augment the sensitivity of *EGFR* gene mutation-positive lung cancer to the EGFR-tyrosine kinase inhibitor (EGFR-TKI), gefitinib. When cocultured with *EGFR* mutation positive lung cancer cells, these CAFs increased the apoptic effect of gefitinib on cancer cells, whereas, in the absence of gefitinib, they did not affect cancer cell viability. The assay using different single cell-derived clones demonstrated that the aforementioned sensitizing ability is clone-specific. Microarray analysis revealed that CD200 was expressed at much higher levels in this CAFs. Knocking down of CD200 expression deprived CAFs of their sensitizing potential, suggesting that CD200 is the functional molecule responsible for the effect. Immunohistochemical analysis of samples from patients receiving postoperative gefitinib treatment revealed that the individuals whose resected lung adenocarcinomas contained CD200-positive CAFs tended to have longer progression free survival of gefitinib when they recurred after surgery. These results suggest that CD200-positive CAFs can augment the sensitivity to EGFR-TKIs and may possess far reaching applications in the therapeutic use of EGFR-TKIs.

In patients with advanced non-small cell lung cancer (NSCLC), epidermal growth factor receptor (*EGFR*) mutations are reportedly a predictive factor for the effect of EGFR tyrosine kinase inhibitors (EGFR-TKIs)^1^,^2^,^3^,^4^,^5^. Compared to standard first line cytotoxic chemotherapy, EGFR-TKIs have been shown to prolong progression-free survival (PFS) in NSCLC patients with *EGFR*-activating mutations, such as exon 19 deletions and L858R point mutations^1^,^2^,^3^,^4^,^5^,^6^,^7^. However, 20% to 30% of patients with tumors harboring *EGFR* mutations exhibit intrinsic resistance to EGFR-TKIs. Moreover, patients who initially respond positively to EGFR-TKI treatment frequently develop resistance to these inhibitors^8^,^9^.

Although the molecular mechanisms underlying acquired resistance have been extensively studied^10^,^11^,^12^, there are only a few reports that provide data regarding the factors that contribute to intrinsic resistance. Ng *et al*. presented data about the relationship between the *BIM*-deletion polymorphism and intrinsic resistance to EGFR-TKIs^13^. Engelman *et al*. and Yano *et al*. reported that the HGF-MET pathway induces resistance to gefitinib^14^,^15^. However, in over 50% of the cases where intrinsic resistance is observed, its causes are unknown^16^.

It is widely accepted that biological characteristics of stromal cells can be key regulators of resistance to anticancer drugs^17^,^18^,^19^,^20^. Many investigators have reported that cancer-associated fibroblasts (CAFs) influence the sensitivity to anticancer drugs via the secretion of extracellular matrix proteins, proteases, cytokines, and growth factors. Straussman *et al*. reported that CAF-secreted HGF induced intrinsic resistance of *BRAF*-mutant melanoma to a RAF inhibitor^17^. Wang *et al*. reported that HGF from stromal fibroblasts confers intrinsic resistance to EGFR-TKIs by activating MET, which restores the phosphorylation of downstream MAPK/extracellular signal-regulated kinases (MAPK-ERK 1/2) and activates the phosphoinositide 3-kinase (PI3K)/Akt signaling pathway in lung adenocarcinomas displaying *EGFR* mutations^21^,^22^. Recently, we reported that podoplanin-positive CAFs induce primary resistance to EGFR-TKIs in lung adenocarcinomas exhibiting *EGFR* mutations, with podoplanin playing a functional role in this effect^23^. Therefore, the mechanisms underlying CAF-induced intrinsic resistance against gefitinib attract significant attention.

Even though CAFs reportedly decrease the antitumor effect of gefitinib, there have been clinical cases where an impressive response to gefitinib was observed^24^. In order to explain this contradiction, we hypothesized that certain types of CAFs might have the ability to augment sensitivity to gefitinib. Here, we report that CAFs isolated from a lung adenocarcinoma patient intensified the antitumor effect of gefitinib on *EGFR* mutation-positive lung adenocarcinoma cells.

## Results

### Antitumor effect of gefitinib on PC9 cells cocultured with CAFs

**Figure 1:**
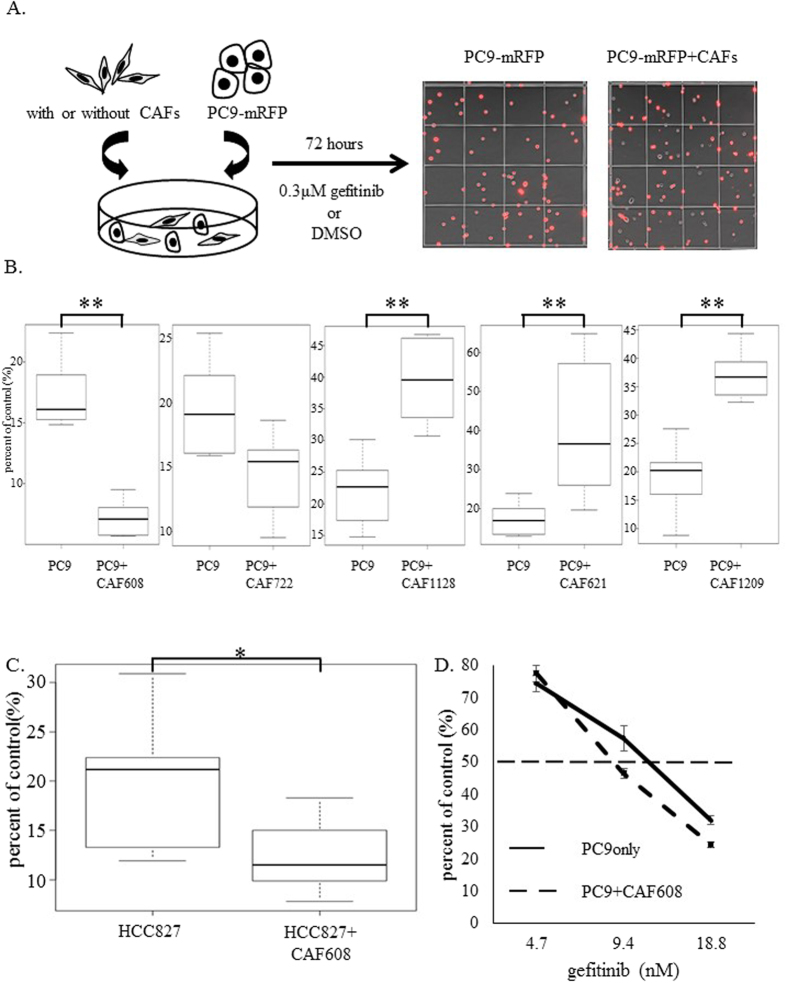
Cocultures of PC9-mRFP cells and CAFs. (**A**) Design of an *in vitro* coculture model. The number of mRFP-positive PC9 cells were calculated 72 h after gefitinib administration. Images of PC9-mRFP cells (red cells) alone or in the presence of CAFs (non-labeled cells) are displayed at the left and right portion, respectively. (**B**) Relative numbers of PC9-mRFP cells cocultured with CAFs from five different patients, after treatment of the cultures with gefitinib. (**C**) Comparison of the percent of control of HCC827-mRFP cells when cultured alone or with CAF608 cells. (**D**) A dose-response study of the effect of gefitinib on the percent of control of PC9-mRFP cells, cultured alone or with CAF608 cells.

Generally, CAFs are believed to promote resistance of *EGFR* mutation-positive lung adenocarcinoma to EGFR-TKIs via soluble factors or direct contact, whereas the expression of these factors varies among CAFs derived from different tumors. We cocultured PC9-mRFP cells with CAFs isolated from five lung adenocarcinoma patients (patients IDs: 608, 621, 722, 1128 and 1209) (Fig. 1A). Experiments with PC9-mRFP cells cocultured with CAF621, 1128 and 1209 gave results consistent with those of previous reports; specifically, the numbers of PC9-mRFP cells in gefitinib-treated cocultures were significantly higher compared to gefitinib treated PC9-mRFP monocultures^21^,^22^. CAF722 did not change the number of viable PC9-mRFP cells after gefitinib treatment. Surprisingly, the number of viable PC9-mRFP cells observed after the administration of gefitinib on cocultures with CAF608 was significantly lower in comparison with gefitinib-treated PC9-mRFP monocultures (7.2% vs 17.2%, P < 0.05) (Fig. 1B). This difference was not observed in the absence of gefitinib treatment (Supplemental Fig. 1A). These findings suggested that CAF 608 cells augment the sensitivity of PC9 cells to gefitinib.

We repeated the assay with mRFP-labeled cells of a different cancer cell line, HCC827 which also harbors an *EGFR* mutation (exon 19 deletion). We obtained similar results (Fig. 1C), thus the sensitivity-enhancing effect of CAF608 cells is no specific to PC9 cells. A dose-effect curve revealed that the presence of CAF608 cells reduced the IC50 of gefitinib for reducing the number of PC9-mRFP cells from 11.7 nM to 8.7 nM (Fig. 1D). Our results suggest that the presence of CAF608 cells makes *EGFR* mutation-positive cell lines more sensitive to gefitinib. It should also be noted that CAF608 cells themselves are not sensitive to gefitinib, as administration of the drug to CAF608 monocultures did not affect the number of CAF608 cells (Supplemental Fig. 1B).

Our next step was to examine whether or not the increased sensitivity of PC9 cells to gefitinib was caused by soluble factors secreted by CAF608 cells. As seen in Supplemental Fig. 2A, the addition of CAF608 culture-derived supernatant to PC9-mRFP cultures not only failed to make cancer cells more sensitive to gefitinib, as was the case in the culture experiments, but actually caused a small increase in the number of viable PC9-mRFP cells compared to gefitinib-treated PC9-mRFP cultures to which no supernatant has been added (21.7% vs. 17.8%) (Supplemental Fig. 2B). In contrast, the effect of CAF621 culture-derived supernatant on the survival rate of PC9-mRFP after gefitinib treatment was similar to the one observed in the coculture experiments, i.e., a significant increase in the number of viable PC9-mRFP cells was observed (35.7% vs. 17.8%, P < 0.05). RT-PCR assay revealed that CAF621 highly expressed HGF, a humoral factor known to enhance resistance against the antitumor effect of gefitinib (Supplemental Fig. 2C). Based on these results, it was expected that intensifying gefitinib sensitivity by CAF608 might be caused by direct cell-cell contacts.

### Measurement of p-Akt and p-ERK1/2

The PI3K/AKT pathway and the MAPK/ERK pathway are associated with the signaling pathways of several receptor tyrosine kinases, such as EGFR, and play a role in the intrinsic resistance to EGFR-TKIs^22^. We evaluated the p-Akt and p-ERK1/2 levels in PC-9 cells, alone or cocultured with CAF608 or CAF621 after gefitinib administration. In this coculture assay, we analyzed the phosphorylation status of PC9-GFP cells after setting the FL1-positive threshold to an appropriate level (Supplemental Fig. 3A and Supplemental Fig. 4). Twenty-four hours after the addition of gefitinib, the levels of p-ERK1/2 in PC9-GFP cells cocultured with CAF621 cells were significantly higher than those of monocultured PC9-GFP cells (average of relative MFI: 0.62 vs. 0.14, P = 0.02). Cocultured PC9-GFP cells also displayed higher levels of p-AKT (0.69 vs. 0.37), but the difference was not statistically significant. On the other hand, MFIs of p-ERK1/2 and p-Akt of PC9-GFP cells cocultured with CAF608 were 0.35 and 0.74 respectively and did not differ statistically significantly compared to the respective values of PC9-GFP monocultures (Supplemental Fig. 3B and C). These findings suggest that the effect of CAF608 on gefitinib sensitivity is not mediated by either the MAPK or the PI3K pathway.

### CAF608 does not affect the cell cycle status of PC9/Fucci2

**Figure 2:**
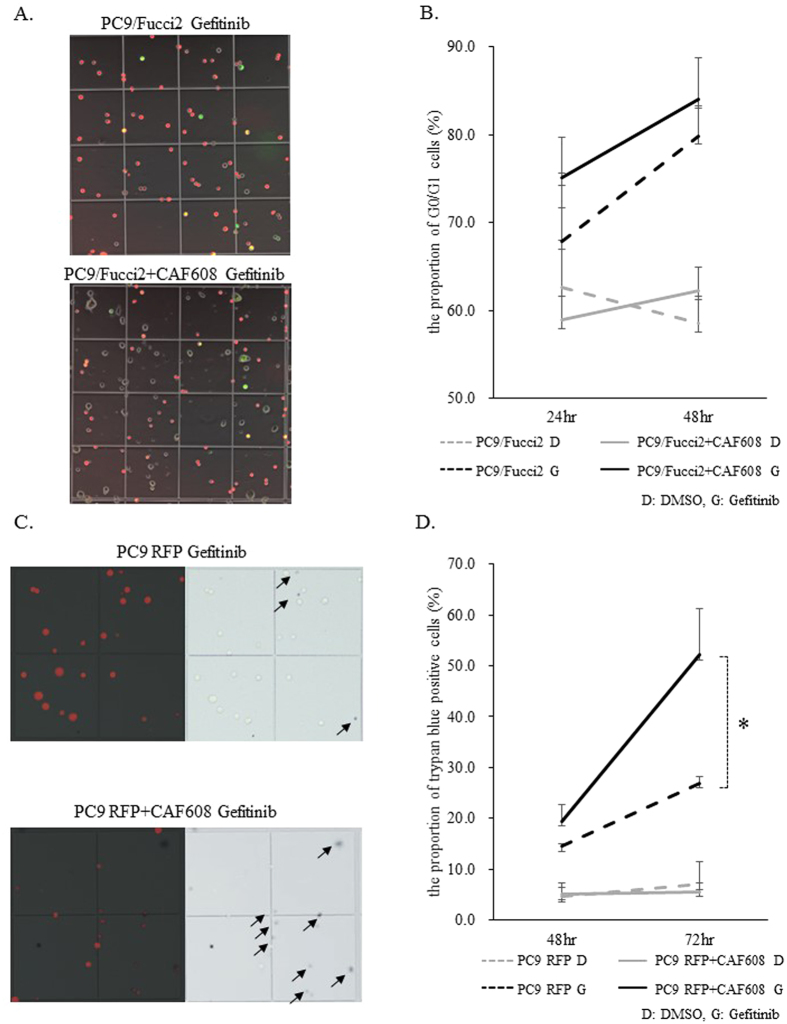
Cell cycle status analysis using PC9/Fucci2 cells, as well as evaluation of the proportion of PC9-mRFP dead cells, in monocultures or cocultures with CAF608. (**A**) Images of PC9/Fucci2 cells in monocultures and cocultures with CAF608. (**B**) The proportion of G0/G1 cells 24 and 48 h after gefitinib administration. (**C**) Images of dead PC9-mRFP cells from monocultures or cocultures with CAF608 after trypan blue staining (trypan blue-positive cells are indicated by arrows). (**D**) The proportion of trypan blue-positive cells in PC9-mRFP monocultures or cocultures of PC9-mRFP and CAF608.

The notable enhancement by CAF608 cells of the gefitinib-induced decrease in the number of viable PC9 cells may be a result of increased cell cycle arrest, increased cell death, or a combination of the two. In order to determine the cause, we added gefitinib to PC9/Fucci2 cells and counted PC9/Fucci2 that were in the G0/G1 or the S/G2/M phases of the cell cycle after 24 and 48 h (Supplemental Fig. 5 and Fig. 2A). The proportion of cells in G0/G1 cells (red fluorescence) in PC9/Fucci2 monocultures gradually increased after addition of gefitinib, with the average at 48 h was increased to 79.9%. The proportion of G0/G1 in PC9/Fucci2 cells in cocultures also increased with time after gefitinib treatment, with the average at 48 hours was 84.1%; there was no statistically significant difference in the G0/G1 proportion between PC9/Fucci2 cells in monocultures and PC9/Fucci2 cells cocultured with CAF608 (Fig. 2B). In the absence of gefitinib, the proportion of G0/G1 PC9/Fucci2 cells was again similar in monocultures and cocultures, which is consistent with the results in Supplemental Fig. 1A.

### CAF608 increased the cell death rate of PC9 cells after gefitinib administration

We calculated the proportion of dead cells 24 and 48 h after gefitinib administration (Fig. 2C). As mentioned above, we have already confirmed that the number of CAF608 is not influenced by gefitinib treatment (Supplemental Fig. 1B). Thus, differences in the number of trypan blue-positive cells between gefitinib-treated cocultures of PC9 with CAF608 cells, and gefitinib-treated monocultures of PC9 cells, can be attributed to differences in the death rate of PC9 cells. Gefitinib treatment causes the proportion of trypan blue-positive cells to increase with time in both PC9-mRFP monocultures and cocultures with CAF608 cells, however the presence of CAF608 cells dramatically increases the death rates. At 72 h, the proportion of dead cells in cocultures was almost twice compared to monocultures (52.2% vs. 26.9%, P = 0.02). However, in the absence of gefitinib, the proportions of dead cells were similar in monocultures and cocultures (the averages at 72 h were 7.1% and 5.7%, respectively) (Fig. 2D). Based on these results, we conclude that CAF608 enhance the effect of gefitinib on PC9 cells mainly through increasing cell death rates, whereas, in the absence of gefitinib, CAF608 cells do not affect the viability of PC9 cells.

### Specific CAF608 clone augments the antitumor effect of gefitinib

**Figure 3:**
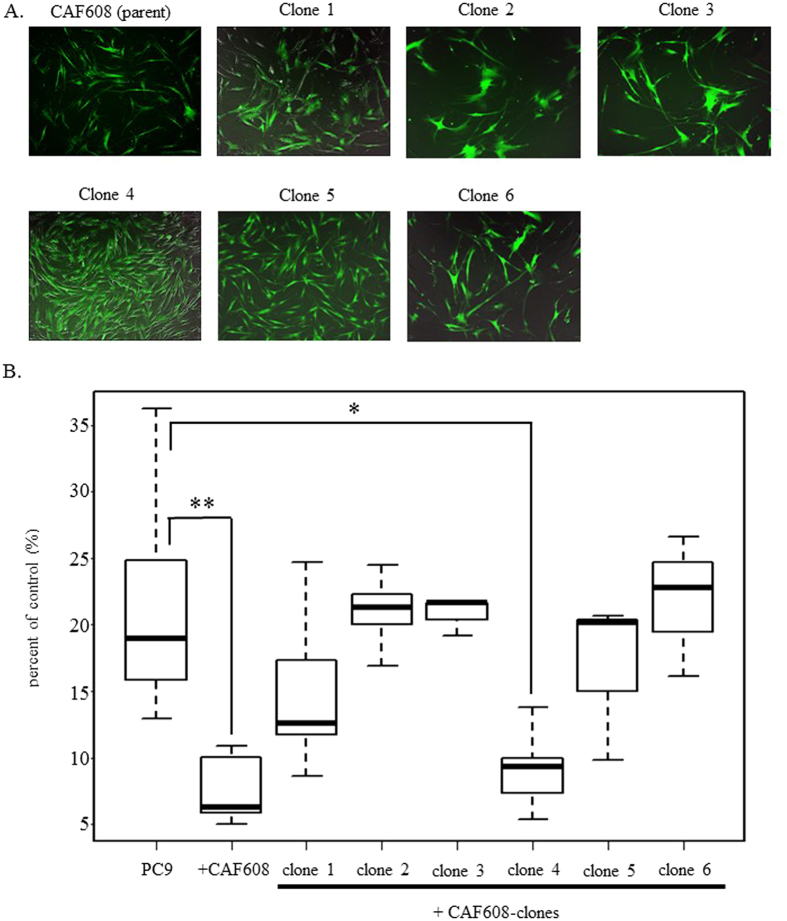
The relative numbers of PC9-mRFP cells in gefitinib-treated monocultures or cocultures with various CAF608 clones. (**A**) Fluorescence images of 6 clones derived from the CAF608 parent population. (**B**) The percent of control of viable PC9-mRFP cells cocultured with various CAF608 clones after gefitinib treatment.

CAFs are generally viewed as a heterogeneous cell populations^25^. In order to support the hypothesis that the effect of CAF608 cells on gefitinib sensitivity is depended on specific CAF608 subpopulations, rather than being a synergistic or additive effect caused by interactions between different clones comprising CAF608, we performed an analysis using single cell-derived CAF608 clones. First, we immortalized CAF608 (parent) by hTERT (Fig. 3A). Next, we tested whether the immortalized CAF608 (parent) were still able to increase the sensitivity of PC9-mRFP to gefitinib. This was confirmed by experiments comparing the number of viable PC9-mRFP after gefitinib treatment of PC9-mRFP monocultures or cocultures with CAF608 cells (6.3% vs. 18.9%, P < 0.05, “PC9” and “+CAF608”). We next created the single cell-derived clones of the immortalized CAF608 cells. As mentioned in the Materials and Methods section, six CAF608 clones were finally established. Each of the six clones was tested with respect to its ability to enhance the sensitivity of PC9-mRFP cells to gefitinib. The number of viable PC9-mRFP cells cocultured with clone 4 after treatment with gefitinib was significantly lower compared with the number of viable PC9-mRFP cells in gefitinib-treated monocultures (9.3% vs. 18.9%, P < 0.05)(Fig. 3B). These results strongly suggest that a specific subpopulation of CAF608 cells is the one with the ability to enhance the sensitivity of PC9 cells to gefitinib.

### CD200 is highly expressed in CAF608 cells

**Figure 4:**
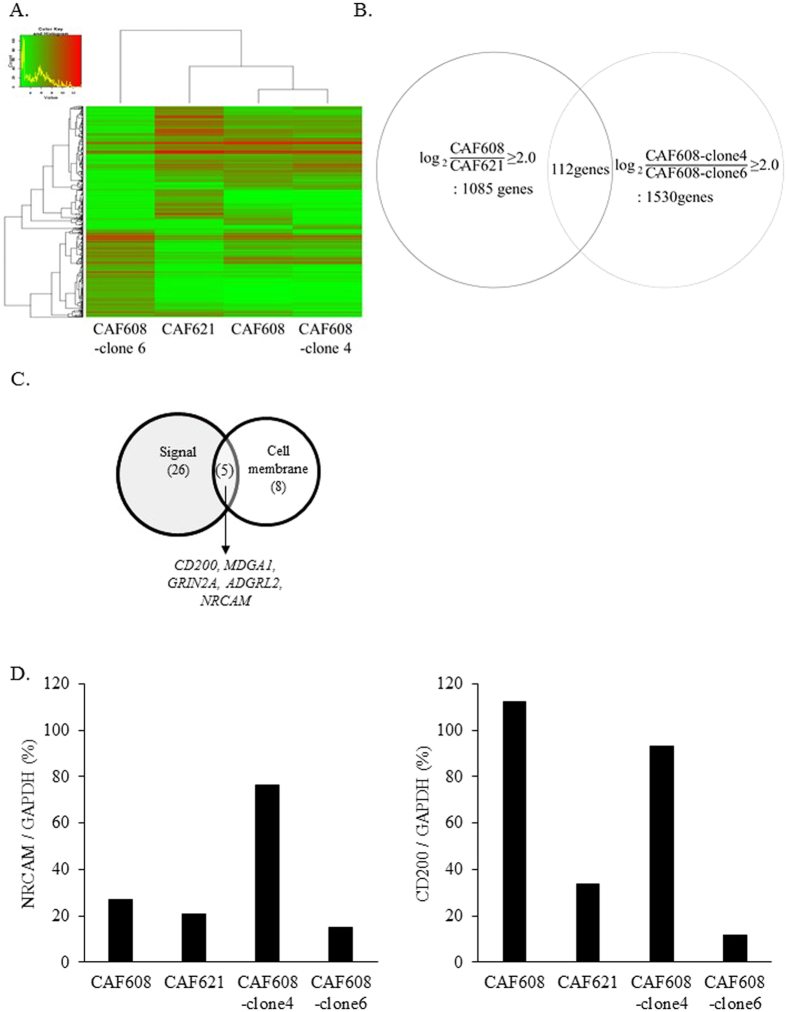
cDNA microarray analysis. (**A**) Heat map plot of CAF608, CAF621, CAF608-clone 4 and CAF608-clone 6. (**B**) Venn diagram showing the number of common genes between the sets of genes whose expression in either the (heterogeneous) CAF608 population or the CAF608-clone 4 was at least four times higher than the expression in CAF621 cells or the CAF608-clone 6, respectively. (**C**) Venn diagram showing the 112 genes after enrichment for two gene ontology terms, “signal” and “cell membrane”. (**D**) Relative *NRCAM* and *CD200* mRNA levels of CAF608, CAF621, CAF608-clone 4 and CAF608-clone 6 cells as determined by RT-PCR assays.

To identify the key molecule, which enhances the antitumor effect of gefitinib, we performed a comprehensive transcriptome analysis using mRNAs from CAF608, CAF621, CAF608-clone 4 and CAF608-clone 6. The cluster analysis of these four groups is shown in Fig. 4A. There were 1085 genes expressed in CAF608 at levels more than 4.0 times (the logarithm of 2 to base 2) higher compared to CAF621. Similarly, we identified 1530 genes that were expressed in CAF608-clone 4 more than 4.0 times higher compared to clone 6. The two lists had 112 common entries, which represent genes highly expressed in both CAF608 parent cells and CAF608-clone4 (Fig. 4B).

Experimental data presented in the first subsection chapter of “Results” suggested that direct contact of CAF608 and PC9 cells is required for the former in increase the sensitivity of the latter to gefitinib (Supplemental Fig. 2). Thus, we used the Database for Annotation, Visualization, and Integrated Discovery (DAVID) Bioinformatics tool^26^,^27^ to filter the information provided by the Gene Ontology database on the 112 genes whose annotations contained the terms “signal” and “cell membrane”. This led to a list of five genes: *CD200, MDGA1, GRIN2A, ADGRL2 and NRCAM* (Fig. 4C). We also chose to focus on *CD200* and *NRCAM*, because these two are reportedly involved in heterotypic cell-cell communication. Our next step was to confirm the microarray data regarding the expression of these 2 genes through RT-PCR. The RT-PCR results regarding the expression levels of *NRCAM* were not consistent with those obtained by the microarray analysis. In contrast, the RT-PCR and microarray results of *CD200* were in agreement (Fig. 4D). Therefore, we decided to focus on the CD200 protein as a candidate molecule.

### CD200 expressed in CAF608 is the functional molecule that augments gefitinib sensitivity

**Figure 5:**
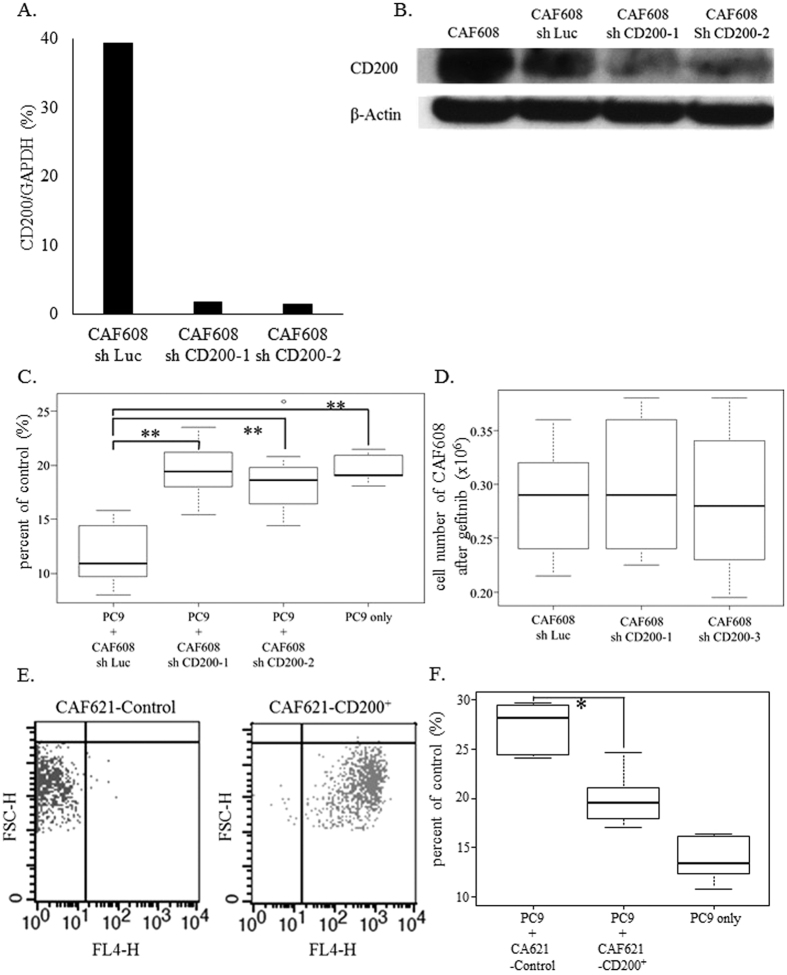
CD200 knockdown and overexpression assays. (**A**) Relative *CD200* mRNA levels of CAF608 cultures transfected with either control vector (sh Luc), sh CD200-1 or -2. (**B**) CD200 protein levels of CAF608, CAF608 sh Luc, sh CD200-1 and -2 cells. (**C**) The percent of control of viable PC9-mRFP cells after gefitinib treatment of cocultures consisting of PC9-mRFP and CAF608 sh Luc, CAF608 sh CD200 -1 or CAF608 sh CD200-2 cells. (**D**) Numbers of CAF608 cells transfected with sh Luc, sh CD200-1 or -2 after gefitinib administration. (**E**) Infection efficacy of lentiviruses as determined by FACS analysis (X axis: FL-4H, Y axis: FSC-H). (**F**) The percent of control of viable PC9-mRFP cells observed after gefitinib treatment of PC9-mRFP cultured alone or cocultured with either CAF621-Control or CAF621-CD200^+^(overexpressing of CD200) cells.

We transfected CAF608 cells with shCD200, creating two CD200-knockdown CAF608 lines, called CAF608 shCD200-1 and CAF608 shCD200-2 (Fig. 5A,B and Supplemental Fig. 6). Gefitinib was administered to cocultures containing PC9-mRFP cells with either CAF608 shCD200-1, CAF608 shCD200-2 or control (CAF608 sh Luc) cells. After gefitinib treatment, the number of PC9-mRFP cells was significantly higher in the cocultures containing either of the two CD200-knockdown CAF608 cells than those containing the CAF608 sh Luc cells. (shCD200-1;19.4% and shCD200-2;18.6% vs. sh Luc;10.9%, P < 0.05, Fig. 5C). On the contrary, the number of CAF608 cells after gefitinib treatment was similar irrespective of the contained vectors (Fig. 5D), indicating that the observed changes in the viability between PC9-mRFP cells cocultured with CD200-knockdown CAF608 cells and PC9-mRFP cells cocultured with control CAF608 sh Luc cells are not caused by CD200-related changes in the viability of the CAF608 cells themselves. We also performed experiments using CAF621 cells overexpressing CD200 (CAF621-CD200^+^, Fig. 5E). After gefitinib treatment, the number of viable PC9-RFP cells was significantly lower when the cancer cells were cocultured with CAF621-CD200^+^ compared to when they were cocultured with control CAF621 cells (19.4% vs. 28.1%, P < 0.05, Fig. 5F). Taking these results into account, we conclude that CD200 expressed by CAF608 cells is the functional molecule responsible for augmenting the sensitivity of cancer cells to gefitinib.

### Lung adenocarcinoma patients with CD200-positive CAFs tend to be associated with a better response to EGFR-TKIs

**Figure 6:**
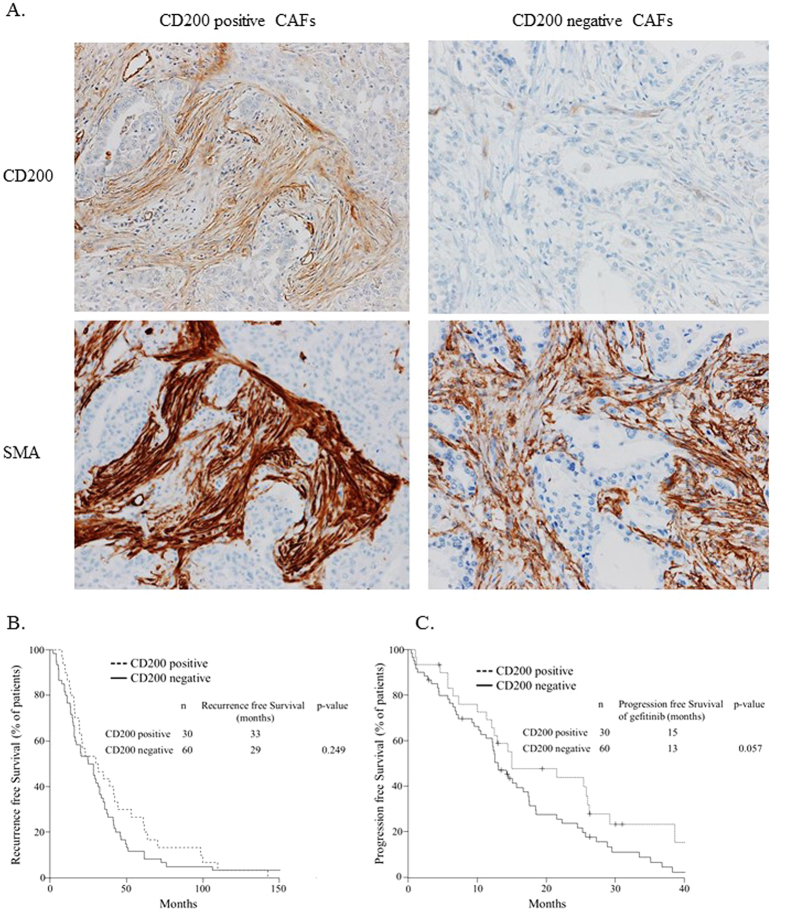
The clinical impact of CD200-positive CAFs in lung adenocarcinoma patients treated with gefitinib. (**A**) Immunohistochemical findings of α-SMA-positive/CD200-positive and α-SMA-positive/CD200-negative CAFs. (**B**) Recurrence free survival of *EGFR* mutation-positive lung adenocarcinoma patients with CD200-positive or CD200-negative CAFs. (**C**) Progression free survival of gefitinib-treated *EGFR* mutation-positive lung adenocarcinoma patients with CD200-positive or CD200-negative CAFs.

If CD200-positive CAFs augment the effect of gefitinib, one would expect the PFS of the patients possessing CD200-positive CAFs in the microenvironment of the tumor to be elongated after treatment. To confirm this hypothesis, we examined a total of 90 patients displaying postoperative recurrence who were being treated with a gefitinib. We divided these patients in two groups based on the expression of CD200 by CAFs (Fig. 6A). Thirty of the patients had CD200-positive CAFs, which were also positive for α-SMA (a marker of CAFs). The proportions of EGFR Exon 19 deletion and L858R were almost equal in both the CD200-positive and the CD200-negative patient group (Supplemental Table 4). About 30 percent of patients in either of the two groups were being administered adjuvant chemotherapy. The majority of patients in both groups had a good performance status. The postoperative recurrence free survival was similar between CD200-positive and negative patients (p = 0.249, Fig. 6B). Conversely, the progression free survival after gefitinib treatment tended to be longer in patients with CD200-positive CAFs compared to patients with CD200-negative CAFs (p = 0.057, Fig. 6C).

## Discussion

The emergence of resistance to EGFR-TKIs through a variety of mechanisms is the most serious problem in their clinical application. Methods for reversing the acquired resistance to EGFR-TKIs have been reported, such as Met kinase inhibitor, Metformin and Anti-HGF antibody^15^,^28^,^29^. However, researchers have largely overlooked the mechanisms underlying intrinsic resistance of cancer cells to EGFR-TKIs^13^,^16^. Furthermore, there are only some reports about the effect of CAFs on intrinsic resistance against EGFR-TKIs^21^,^23^, both of which report CAFs as a factor that makes cancer cells more resistant to these inhibitors. Therefore, our discovery of a subpopulation of CAFs that actually makes cancer cells more sensitive to gefitinib is a novel finding and reveals the diverse effects of CAFs on targeted cancer therapy. It is well known that CAFs play important roles in several aspects of tumor progression as well as in the chemotherapeutic process. However, CAFs derived from different patients exhibit different biological properties. Furthermore, even CAFs isolated from a single patient are a heterogeneous cell population in terms of functional potential^25^. The current study clearly demonstrates that CAFs consist of subpopulations that can differ in their effects on cancer cell sensitivity to EGFR-TKIs. We could not observe the augmentation effect by fibroblasts which are derived from the normal alveolar lesion of lung in the same patient as CAF608 (data not shown). Therefore our study demonstrates that the augmentation effect is the unique in CAF608. Our results suggest that CAFs should be divided into two categories with respect to their effect on cancer cell sensitivity; drug resistance-promoting and drug sensitivity-promoting phenotypes. CAF608 cells, which are studied in the current report, would obviously be classified into the second variety.

Through a process that included microarrays, bioinformatics analysis of results, and RT-PCR validation, we chose to focus on *CD200*, a membrane protein that is expressed in both the heterogeneous CAF608 population and its CAF608 clone 4 at levels much higher than in CAF621 cells. Experiments in which the *CD200* gene was knocked down or overexpressed confirmed that CD200 is the protein that augments the antitumor effect of gefitinib. Moreover, the percentage of viable PC9 cells after gefitinib treatment in the presence of 1 μM (24.6%) or 10 μM (24.8%) of recombinant human CD200 was statistically significantly lower (P < 0.05 for both concentrations) compared to control cultures to which recombinant GFP had been added at a final concentration of 1 μM (28.3%) or 10 μM (32.5%) (Supplemental Fig. 7). Immunohistochemical analysis also demonstrated that lung cancer tissue containing CD200-positive CAFs tends to be more sensitive to gefitinib compared to tissue containing CD200-negative cell. CD200, also known as OX2 membrane glycoprotein, is normally expressed in neurons, hair follicle epithelial cells, lymphocytes and mesenchymal stem cells^30^. The interaction of CD200 with its receptor (CD200R1), which is expressed in macrophages, lymphocytes and monocytes triggers an immunosuppressive response that inhibits macrophage activity^31^. However, we did not detect CD200R expression in PC9 cells (data not shown). This suggests that CD200 exerts by interacting with a yet unknown receptor of cancer cells, whose activation triggers a pro-apoptotic signal cascade only upon EGFR-TKI treatment, through a pathway distinct from PI3K-Akt and/or ERK signaling. Alternatively, CD200 may induce a conformational change in other associated molecules, resulting in pro-apoptotic signal transduction in tumor cells.

*EGFR* mutation-positive cell lines cocultured with CAF608 without gefitinib did not display changes in the number of dead cells, whereas the presence of gefitinib allows CAF608 cells to act as gefitinib sensitizers. These results support the introduction of a new treatment strategy against *EGFR* mutation positive NSCLC. Treatment with a combination of gefitinib and a cancer cell gefitinib sensitizer tailored according to how CAF608 exert this role may provide a better therapeutic result compared to administrations of gefitinib alone. Ramirez *et al*. revealed the presence of “drug-tolerant cancer persister cells” which are small subpopulations of cancer cells that evade strong selective drug pressure. They demonstrated the diversity of drug resistance mechanism arising from these cells by using an erlotinib-resistant cell line^32^. After a period of negligible growth, a small fraction of persister cells gain the ability to expand. Thus, cancer cell survivors such as drug-tolerant cancer persister cells are considered one of the causes of acquired resistance. Targeting the survival and expansion of drug-tolerant cancer persister cells could be a powerful strategy for decreasing the chance of subsequent acquisition of genetic alterations. As CD200 maximizes the effect of gefitinib, it could reduce the number of drug-tolerant cancer persister cells. It would be interesting to examine the possibility whether CAF608-iuduced sensitivity is also found in another TKI or another driver oncogene mutation positive lung cancer cell lines. We consider CD200-mediated CAF-cancer cell crosstalk to be a promising candidate for the development of novel treatment strategies against acquired resistance.

## Materials and Methods

### Clinical samples

All specimens were collected after obtaining written comprehensive informed consent from the patients. The study was conducted with the approval of the Institutional Review Boards of the National Cancer Center. IRB approval number of this study is 2014-070. All methods were carried out in accordance with the approved guidelines.

### Cell culture and reagents

The *EGFR* mutant human lung adenocarcinoma cell lines PC-9 (del E746_A750) and HCC-827 (del E746_A750) were purchased from the European Collection of Cell Culture and the American Type Culture Collection. The PC-9 and HCC827 cell lines were maintained in RPMI 1640 supplemented with 10% heat-inactivated fetal bovine serum (FBS; Sigma, St. Louis, MO), 1% glutamine, and antibiotics (1% penicillin and streptomycin; Sigma). The cultures were incubated at 37 °C in an atmosphere containing 5% CO_2_. Gefitinib was obtained from Tocris Bioscience (Ellisville, MO).

CAFs were prepared from human lung cancer tissues as previously reported^33^,^34^. The characteristics of the adenocarcinoma cases from which primary CAFs were obtained are shown in Supplemental Table 1.

### Evaluation of the antitumor effect of gefitinib

PC9 (4.0 × 10^5^ cells) or HCC827 (4.0 × 10^5^ cells), labeled with monomeric red fluorescent protein (mRFP), were cocultured with an equal number of CAFs in 10 cm dishes as described previously^23^. Gefitinib (0.3 μM, final concentration) or DMSO was added the next day. After 72 h, we counted the PC9-mRFP or HCC827-mRFP cells using C-Chip disposable hemocytometers (Digital Bio, Seoul, South Korea) with an BZ-9000 inverted-type fluorescence microscope (Keyence, Japan) (Fig. 1A). The effect of gefitinib is evaluated by dividing the number of labeled cells after treatment with the inhibitor, with the number of cells in the control cultures (which were treated with DMSO), according to formula.


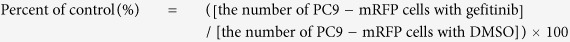


For practical reasons, henceforth this value is referred to as “percent of control” instead of “number of cells after treatment with the inhibitor expressed as a percentage of the number of cells observed after treatment with DMSO”.

### Collection of condition medium

PC9-mRFP, CAF608 or CAF621 cells were seeded in 10 cm dishes (1.0 × 10^6^ cells/dish) and incubated for 24 h. The supernatants of the CAF608 and CAF621 cultures were collected, filtered using Steri-flip filter units (Merck Millipore, Billerica, MA, USA) and added to PC9-mRFP cells.

### Real-time reverse transcriptase-polymerase chain reaction (RT-PCR)

Cells were washed with PBS and suspended in 1 ml of TRIzol (Invitrogen), then stored at −80 °C. Total RNA was purified from thawed samples using standard techniques, and cDNA was synthesized using the PrimeScript RT reagent Kit (TaKaRa), according to the manufacturer’s instructions. RT-PCR was performed in a Smart Cycler System (TaKaRa) using SYBR Premix Ex Taq (TaKaRa) and Real time PCR Primers.

Informations of primers used were shown in Supplemental Table 2.

### Measurement of p-Akt and p-ERK1/2

Measurement of phosphorylated-Akt and ERK1/2 of PC9 cells was performed as previously reported^23^. Briefly, PC9 labeled with Green Fluorescent Protein (GFP) and CAFs were plated in 10 cm dishes one day before gefitinib treatment. Twenty-four hours after the addition of gefitinib, cells were fixed with the addition of BD Phosflow Fix Buffer (BD Biosciences, Great Lakes, NJ, USA). After 15 min incubation at room temperature (RT), cells were permeabilized in ice-cold BD Perm Buffer III (prechilled at −20 °C) for 30 minutes. The samples were washed with BD statin buffer and stained for 1 h with conjugated antibodies recognizing p-Akt (Ser473) (Alexa Fluor^®^ 647; Cell Signaling Technology), p-ERK1/2 (Thr202/Tyr204) (Alexa Fluor^®^ 647; Cell Signaling Technology), as well as an IgG isotype control (BD Biosciences). We selected a region with GFP-positive cells as cancer cells for analysis. The relative mean fluorescence intensity (MFI) of p-Akt and p-ERK was defined as the MFI of each group divided by the MFI of PC9 cells without gefitinib treatment.

### Generation of PC9/Fucci2 cells

The cell cycle was visualized using the Fucci2 system^35^. Lentiviruses were produced using 293T cells transfected with PCAG-HIV, pCMV-VSV-G-RSV-Rev (RIKEN BioResource Center, Tsukuba, Japan), and Fucci vectors (kindly provided by Dr. A. Miyawaki of the RIKEN Center). Transfection was achieved using the Lipofectamine 2000 reagent (Invitrogen, Carlsbad, CA, USA), according to the manufacturer’s instructions. Vector-containing medium was filtered through a 0.45 μm filter, and 8 μg/mL of polybrene (Santa Cruz Biotechnology, Dallas, TX, USA) was added to increase the efficiency of transduction. First, we transfected mCherry-hCdt1(30/120)/pCSII-EF-MCS into PC9 cells. The mCherry-positive cells were selected through FACS (FACSAria, BD Biosciences) and then transduced with Venus-hGeminin(1/110)/pCSII-EF-MCS. Venus-positive cells were sorted and named PC9/Fucci2. Thereafter, PC9/Fucci2 cells and samples of cells from each stage of their generation were fixed and stained with DRAQ5 (BioStatus, Shepshed, UK). DNA contents of each fraction were analyzed by FACS (FACS caliber, BD Biosciences) and histograms were merged. The results confirmed that PC9/Fucci2 cells display the same distribution as each PC9 fraction produced during their generation.

### Evaluation of the proportion of G0/G1 cells using PC9/Fucci2 cells

PC9/Fucci2 cells (1.4 × 10^5^ cells/dish) were cocultured with an equal number of CAF608 cells in 6 cm dishes. At two time points (24 and 48 h) after administration of 0.3 μM gefitinib, we counted the mCherry positive cells and Venus positive cells. The proportion of G0/G1 positive PC-9 cells was calculated according to formula.


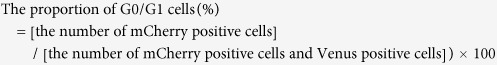


### Evaluation of the number of PC9 dead cells by Trypan blue staining

PC9-mRFP cells were cocultured with CAF608 in 6 cm dishes. Samples were collected (both floating and adherent cells) 48 and 72 h after administration of 0.3 μM gefitinib, and stained with a 0.4% Trypan blue solution. The proportion of Trypan blue-positive cells was calculated with the following formula.


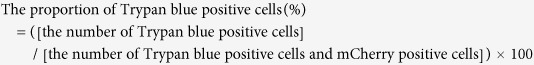


### Generation of CAF608 clones

Generation of CAF608 clones was performed as previously reported^36^. Briefly, single CAF608 cells expressing hTERT-Venus were sorted and seeded in 384-well plates. Fluorescence images were obtained every 12 h using the IncuCyte Live-Cell Imaging System (Essen Bioscience, Ann Arbor, MI, USA). After clones reached semi-confluence, they were replated on 96-well plates. We obtained a total of 6 single-cell-derived clones.

### Evaluation of the antitumor effect of gefitinib using PC9-mRFP cells cocultured with CAF608 clones

PC9-mRFP cells (2.0 × 10^4^ cells/well) were cocultured with an equal number of cells of one of each of the six CAF608 clones in 12-well plates (BD Falcon, BD Biosciences). PC9-mRFP cells were counted by flow cytometry as previously described^23^ at 72 h after gefitinib administration.

### Microarray analysis

Total RNAs from cultured CAF608, CAF621, CAF608-clone 4 and CAF608-clone 6 were isolated using RNeasy Mini kit (250) (QIAGEN). Cyanine-3 (Cy3) labeled cRNA was prepared from 0.1 ug Total RNA using the Low Input Quick Amp Labeling Kit (Agilent) according to the manufacturer’s instructions, followed by RNeasy column purification (QIAGEN, Valencia, CA). Dye incorporation and cRNA yield were checked with the NanoDrop ND-2000 Spectrophotometer. The 0.6 ug of Cy3-labelled cRNA was fragmented at 60 °C for 30 minutes in a reaction volume of 25 ul containing 1× Agilent fragmentation buffer and 2× Agilent blocking agent following the manufacturer’s instructions. On completion of the fragmentation reaction, 25 ul of 2× Agilent hybridization buffer was added to the fragmentation mixture and hybridized to Agilent SurePrint G3 Human GE 8 × 60 K v2 Microarrays for 17 hours at 65 °C in a rotating Agilent hybridization oven. After hybridization, microarrays were washed 1 minute at room temperature with GE Wash Buffer 1 (Agilent) and 1 minute with 37 °C GE Wash buffer 2 (Agilent), then dried immediately. We scanned the slides immediately after washing on the Agilent SureScan Microarray Scanner (G2600D) using one color scan setting for 8 × 60 K array slides (Scan Area 61 × 21.6 mm, Scan resolution 3 um, Dye channel is set to Green PMT is set to 100%). The scanned images were analyzed with Feature Extraction Software 11.5.1.1(Agilent) using default parameters to obtain background subtracted and spatially detrended Processed Signal intensities (TaKaRa).

### CD200 knockdown and overexpression studies

The oligonucleotides that were chemically synthesized for the CD200 short hairpin RNA (shRNA) experiments are displayed in Supplemental Table 3. To create entry clones, the top and bottom strands of the oligonucleotide were annealed and ligated into pENTR4-H1. Thereafter, a LR recombination reaction was performed between the entry clones and CS-RfA-EG using Gateway LR Clonase (Thermo Fisher Scientific, Waltham, MA, USA) according to the manufacturer’s instructions. Both pENTR4-H1 and CS-RfA-EG were obtained from the RIKEN BioResource Center.

For the CD200 overexpression experiment, a lentivirus expression vector for human CD200 was generated by subcloning pUNO1-hCD200 (Invitrogen) into the CSII-CMV-RfA-IRES2-Venus vector.

The lentiviruses were produced using 293T cells previously prescribed^37^. Transfection was performed using the Lipofectamine 2000 reagent (Invitrogen) according to the manufacturer’s instructions.

### Western Blotting

Cells were lysed in whole-cell extraction buffer (20 mM HEPES-NaOH, 0.5% NP-40, 15% glycerol) containing a complete protease inhibitor cocktail (Roche, Basel, Switzerland). The proteins were separated in a 12% SDS-polyacrylamide gel and transferred to an Immobilon-P PVDF membrane (Merck Millipore). Blots were incubated for 1 h at RT with a mouse monoclonal antibody recognizing human CD200 (Proteintech, Rosemont, IL, USA) or a mouse polyclonal antibody recognizing human β actin (Santa Cruz). After washing in TBS-T, the membranes were incubated for 1 h at RT with horseradish peroxidase-linked anti-mouse IgG (GE healthcare, Chicago, IL, USA). ECL Western Blotting Detection Reagents (GE Healthcare) were used to develop the high-performance chemiluminescence film (GE Healthcare).

### Patients included in this study

We selected 90 postoperative recurrent patients with lung adenocarcinoma harboring an *EGFR*-activating mutation that had been treated with gefitinib at our institution, in the period between January 2002 and December 2012. We identified mutations causing activation of *EGFR* in our patient pool (deletions in exon19, L858R point mutation, L861Q point mutation, and G719 missense point mutations) either through direct sequencing using a Cycleave PCR (SRL Diagnostics, NewDelhi, India), or the peptide nucleic acid-locked nucleic acid (PNA-LNA) PCR-clamp method (LSI Medience, Tokyo, Japan), or a PCR-Invader Assay (Third Wave Technologies, WI, USA). We performed a clinical evaluation of PFS lasting from the initiation of gefitinib to the earliest identifiable sign of disease progression, as determined by evaluation data from CT or MRI imaging according to the RECIST (Response Evaluation Criteria In Solid Tumors) guidelines, or until occurrence of death from any cause.

### Immunohistochemistry

Immunohistochemistry was performed using the BenchMark ULTRA system (Ventana Medical Systems, Tucson, Arizona, USA). Tissue sections were stained with antibody recognizing human CD200 (R&D systems, Minneapolis, MN, USA) or a monoclonal mouse antibody recognizing human smooth muscle actin (DAKO, Carpenteria, CA, USA). According to a previously reported definition^38^, we identified CAFs as spindle-shaped cells which were arranged in disarray fashion within the cancer stroma. A specimen was considered to be positive if at least 5% of the fibroblasts showed an unequivocal positive reaction for CD200.

### Statistical analysis

We performed two-sided Mann-Whitney U tests and Kruskal-Wallis nonparametric tests to analyze our data. The recurrent free survival and progression free survival were estimated using the Kaplan-Meier method, and differences in the variables were calculated using the log-rank test. A *P* value lower than 0.05 was considered statistically significant, unless stated otherwise. All statistical analyses were performed with EZR (Saitama Medical Center of the Jichi Medical University, Shimotsuke, Japan), which is a graphical user interface for the R programming language that was developed by the R Foundation for Statistical Computing^39^. More precisely, EZR is a modified version of R-commander (Free Access), created by importing statistical functions frequently used in biostatistics into the latter.

## Additional Information

**How to cite this article:** Ishibashi, M. *et al*. CD200-positive cancer associated fibroblasts augment the sensitivity of Epidermal Growth Factor Receptor mutation-positive lung adenocarcinomas to EGFR Tyrosine kinase inhibitors. *Sci. Rep.* **7**, 46662; doi: 10.1038/srep46662 (2017).

**Publisher's note:** Springer Nature remains neutral with regard to jurisdictional claims in published maps and institutional affiliations.

## Supplementary Material

Supplementary Information
