# Supplementary material for: CD200-positive cancer associated fibroblasts augment the sensitivity of Epidermal Growth Factor Receptor mutation-positive lung adenocarcinomas to EGFR Tyrosine kinase inhibitors
Source: Sci Rep. 2017 Apr 21;7:46662. doi: 10.1038/srep46662 (PMC5399371; doi:10.1038/srep46662)
Supplement: Supplementary Information [file srep46662-s1.pdf]

Supplementary material for the manuscript

Title: CD200-positive cancer associated fibroblasts augment the sensitivity of Epidermal Growth Factor Receptor mutation-positive lung adenocarcinomas to EGFR Tyrosine kinase inhibitors.

Authors: Masayuki Ishibashi, Shinya Neri, Hiroko Hashimoto, Tomoyuki Miyashita, Tatsuya Yoshida, Yuka Nakamura, Hibiki Udagawa, Keisuke Kirita, Shingo Matsumoto, Shigeki Umemura, Kiyotaka Yoh, Seiji Niho, Masahiro Tsuboi, Kenkichi Masutomi, Koichi Goto, Atsushi Ochiai, *Genichiro Ishii

**Supplemental Figure legends**

Supplemental Figure 1; *Supplemental data of Figure 1*

A. Numbers of PC9-mRFP cells cultured alone or cocultured with CAFs in the absence of gefitinib.

B. The number of CAF608 cells 72h after administration of gefitinib or DMSO.

Supplemental Figure 2; *The effect of CAF culture supernatant on the sensitivity of PC9-mRFP cells to gefitinib*

A. Scheme of the treatment model. After 72 hours from gefitinib administration, the number of mRFP positive cells (PC9) was calculated.

B. The relative numbers of PC9-mRFP cells after administration of gefitinib to PC9-mRFP cultures treated with supernatant from CAF608 or CAF621 cultures.

C. The relative mRNA levels of *HGF* in CAF608 and CAF621 cultures.

Supplemental Figure 3; Levels of p-Akt and p-ERK1/2 in PC9-GFP cocultured with CAFs

A. The histograms of pAKT and pERK in PC9-GFP cells cultured alone or cocultured with CAFs, after treatment with gefitinib or DMSO.

B. The relative MFIs(mean fluorescence intensities) of pAKT.

C. The relative MFIs of pERK.

Supplemental Figure 4; *The distribution of PC9-GFP cells as determined by FACS analysis*

Non-labeled PC9 cells are non- fluorescent. Almost all PC9-GFP cells are FL-1 positive. In the histograms of PC9-GFP cells cocultured with CAFs, the left region (low FL-1) corresponds to CAFs (X axis: FL-1, Y axis: SSC-H)..

Supplemental Figure 5; *Generation of PC9/Fucci2 cells*
A. Characterization of PC9/Fucci2 cells. Cells showing red (mCherry-positive and mVenus-negative), yellow (mCherry and mVenus-positive), and green (mCherry-negative and mVenus-positive) fluorescence were collected.
B. DNA contents from PC9/Fucci2 cells and cells from the various stages of their generation were stained with DRAQ5 and measured.

Supplemental Figure 6; *Knockdown assay*
The infection efficacy of lentiviruses was evaluated by FACS analysis (X axis: FL-1, Y axis: SSC-H).

Supplemental Figure 7; *Evaluation of the antitumor effect of gefitinib on PC9 cells in the presence of recombinant human CD200*
Non-labeled PC9 cells ($2.2 \times 10^3$ cells/wells) were cultured in 96-well plates (Falcon MICRO TEST 96, BD Biosciences). Recombinant human CD200 Fc chimera protein (R&D Systems) or GFP were added to the cells at a final concentration of 1μM or 10μM. Gefitinib was administered at a concentration of 0.3μM. After 72h, 10μL of CCK8 (Cell Counting Kit 8; Dojindo Laboratoties, Kumamoto, Japan) were added to each well and incubated for 2h. The plates were analyzed using a microplate reader at 450nm. The antitumor effect of gefitinib was evaluated according to the following formula.
the rate of survived cells (%)=([the O.D.450nm of PC9 cells after gefitinib]/[the O.D.450nm of PC9 cells after DMSO]) × 100

Supplemental figure 1

A.

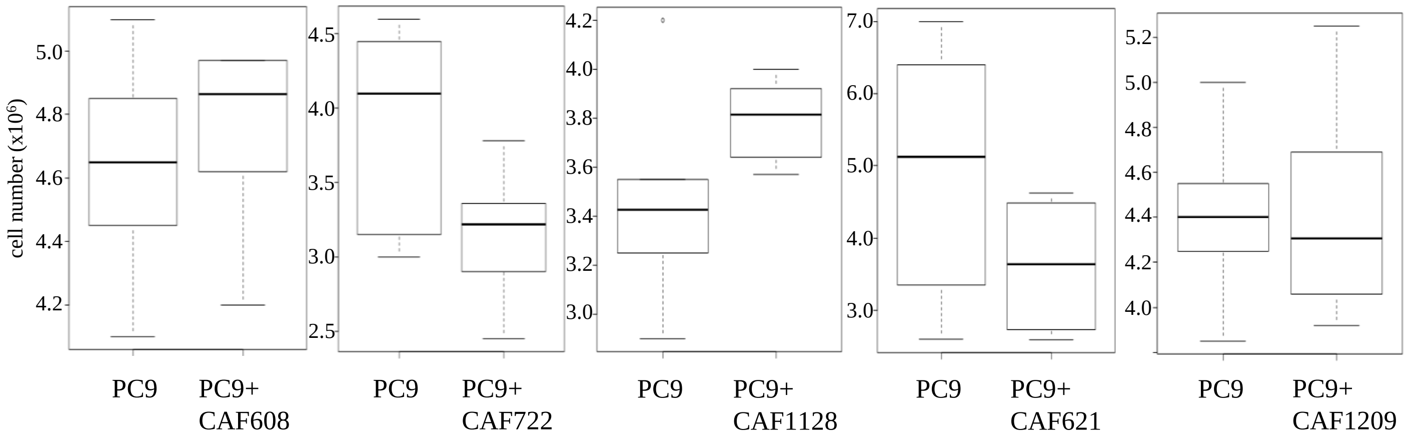


B.

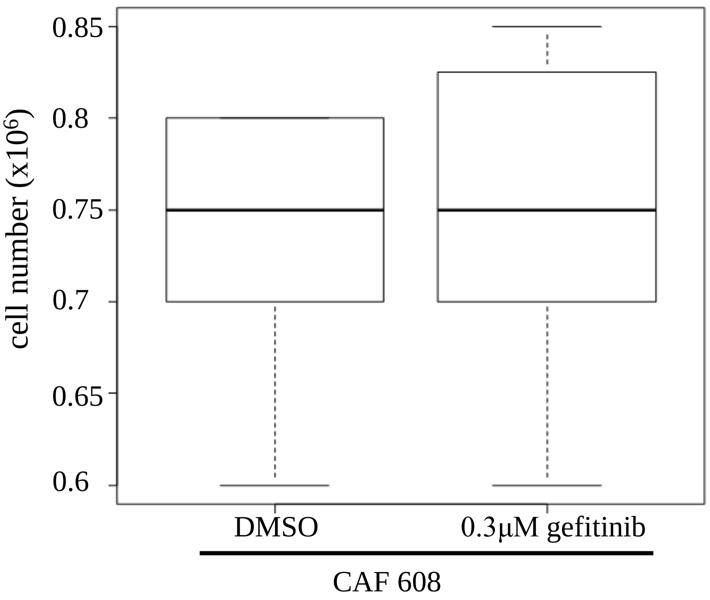

Supplemental figure 2

A.

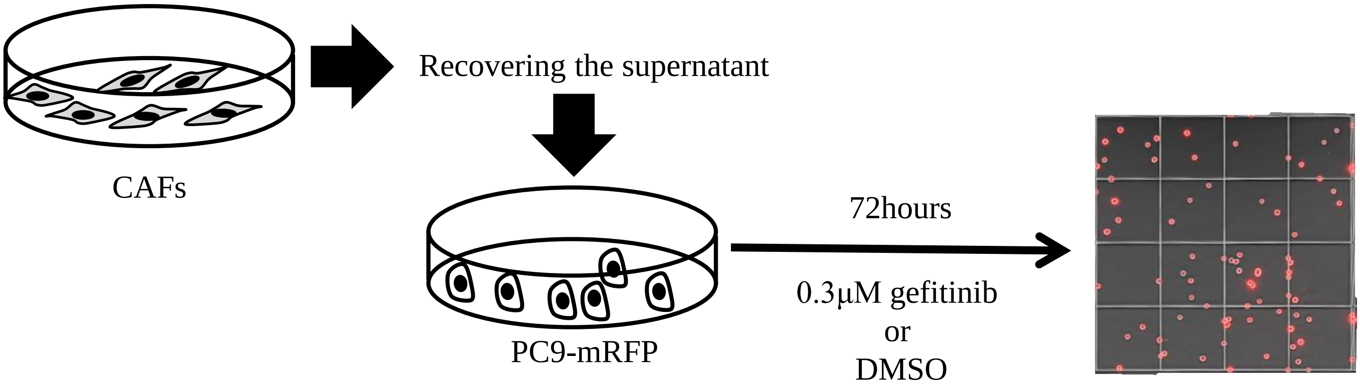


B.

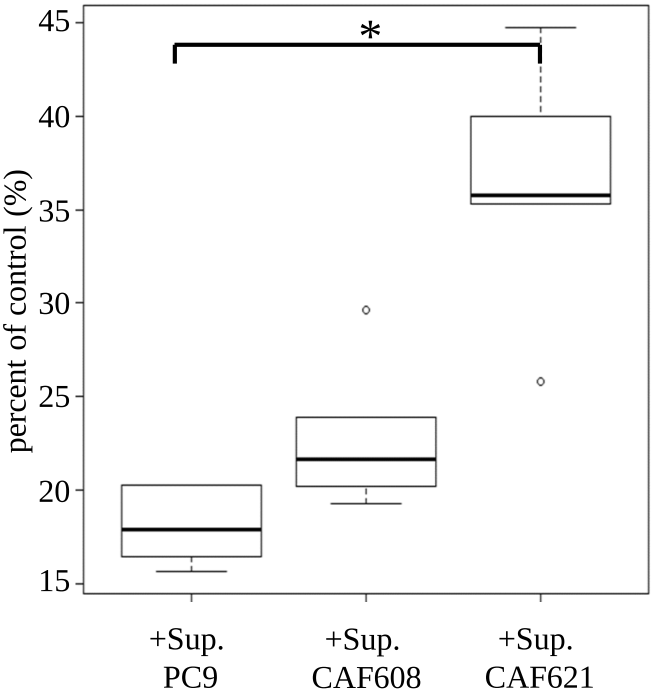


C.

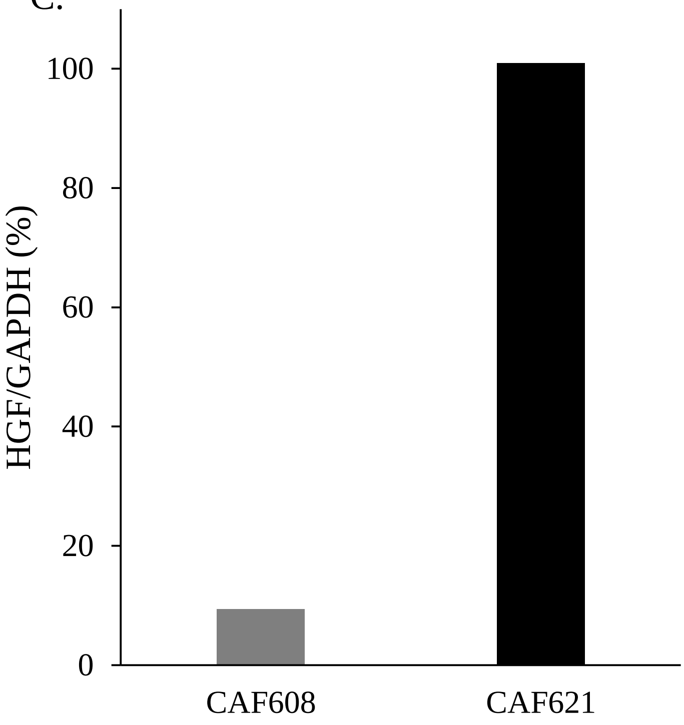

Supplemental figure 3

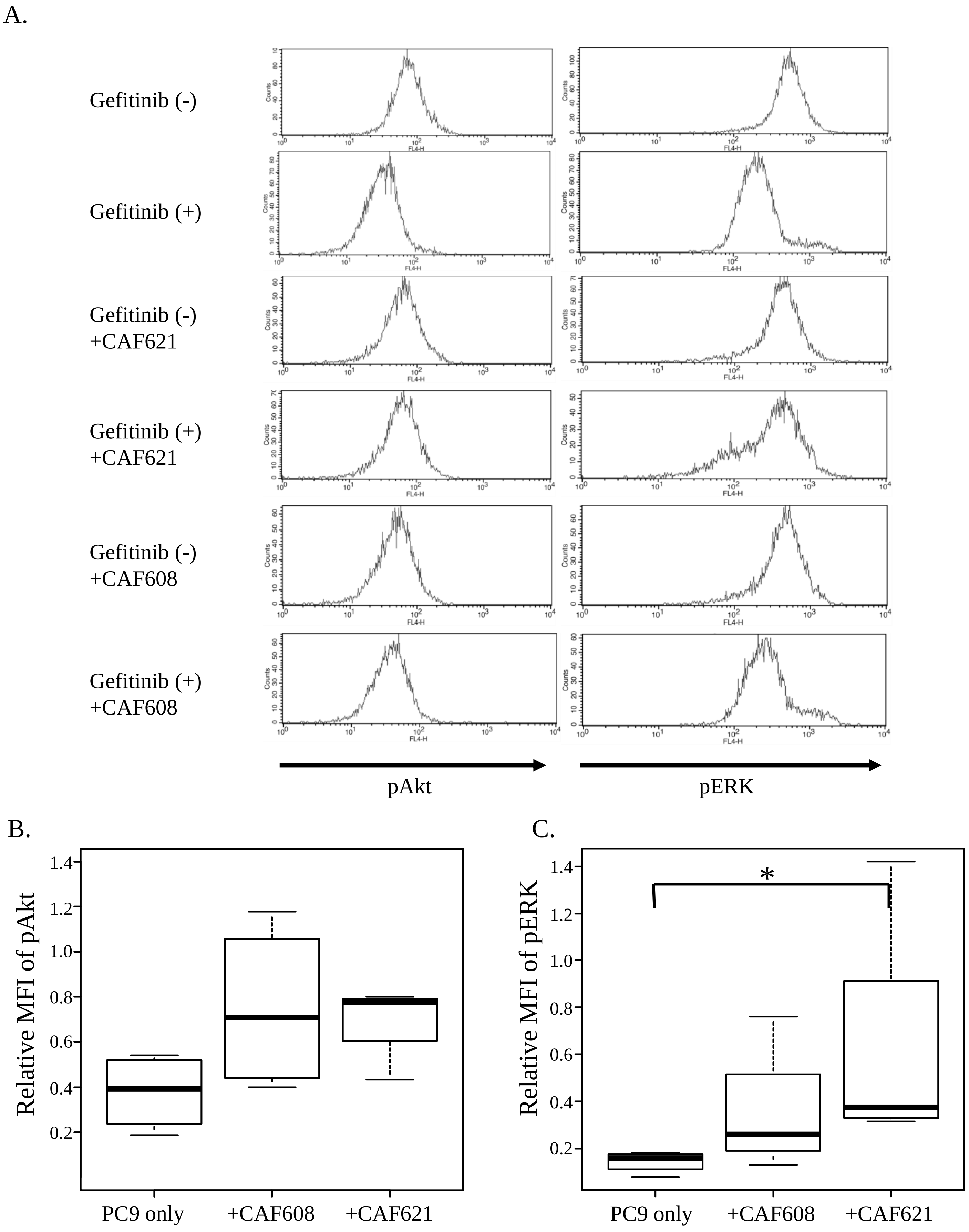

Supplemental Figure 4

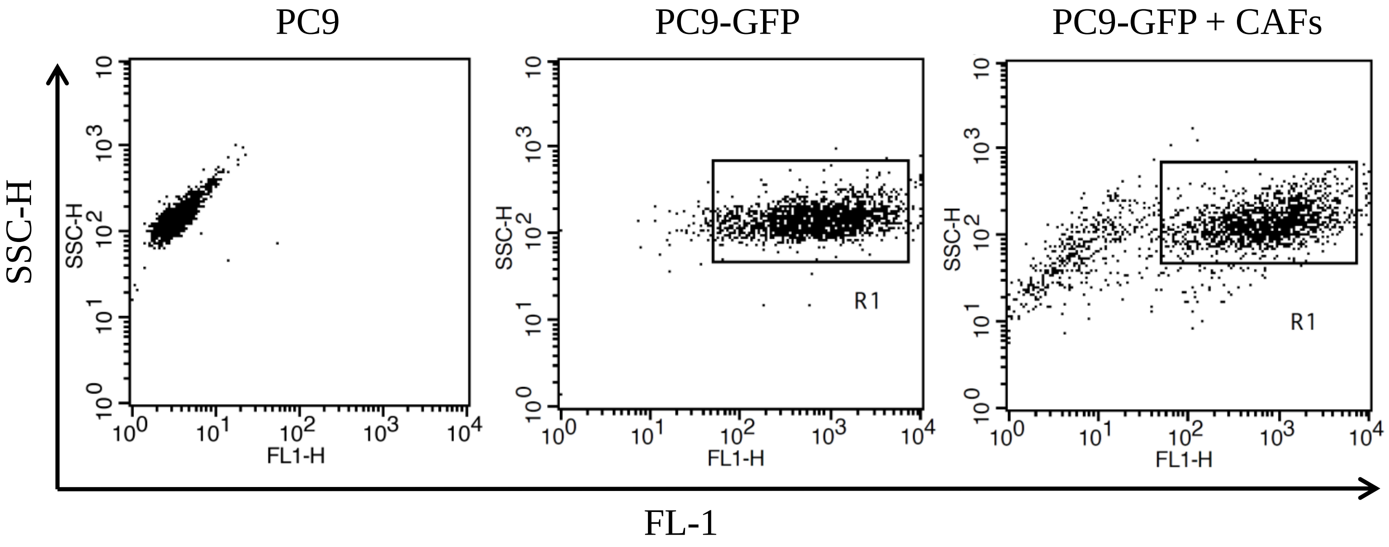

Supplemental Figure 5

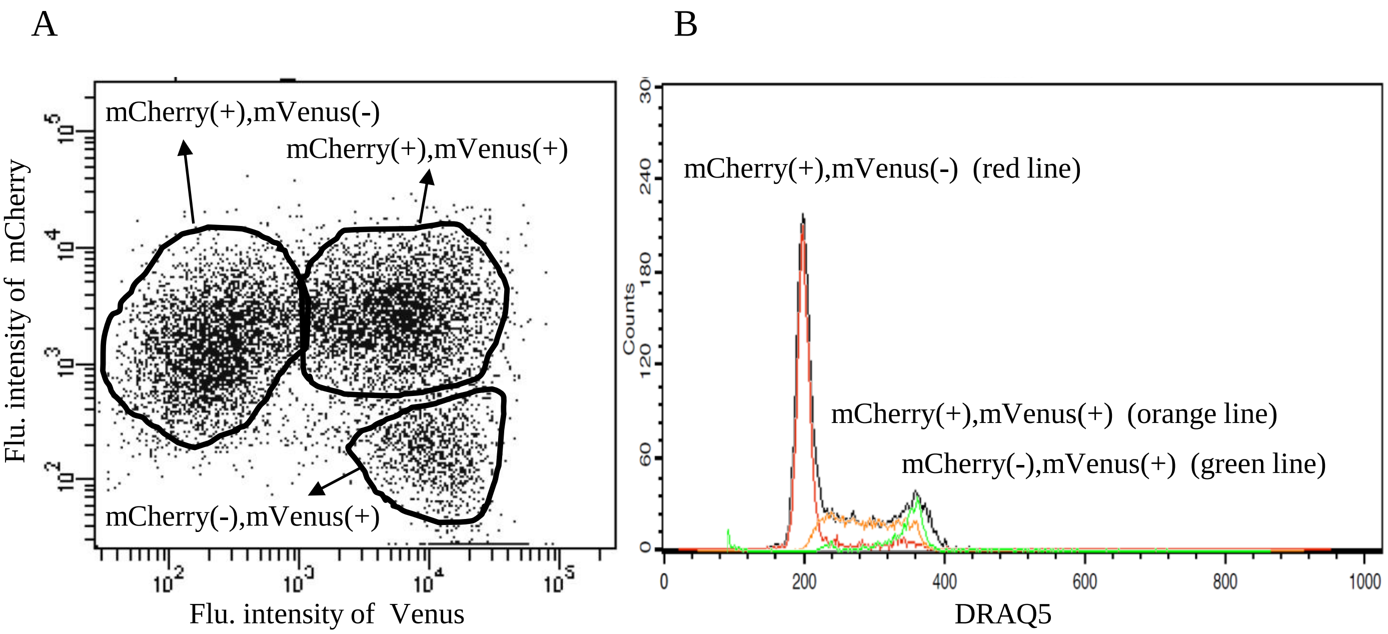

Supplemental Figure 6

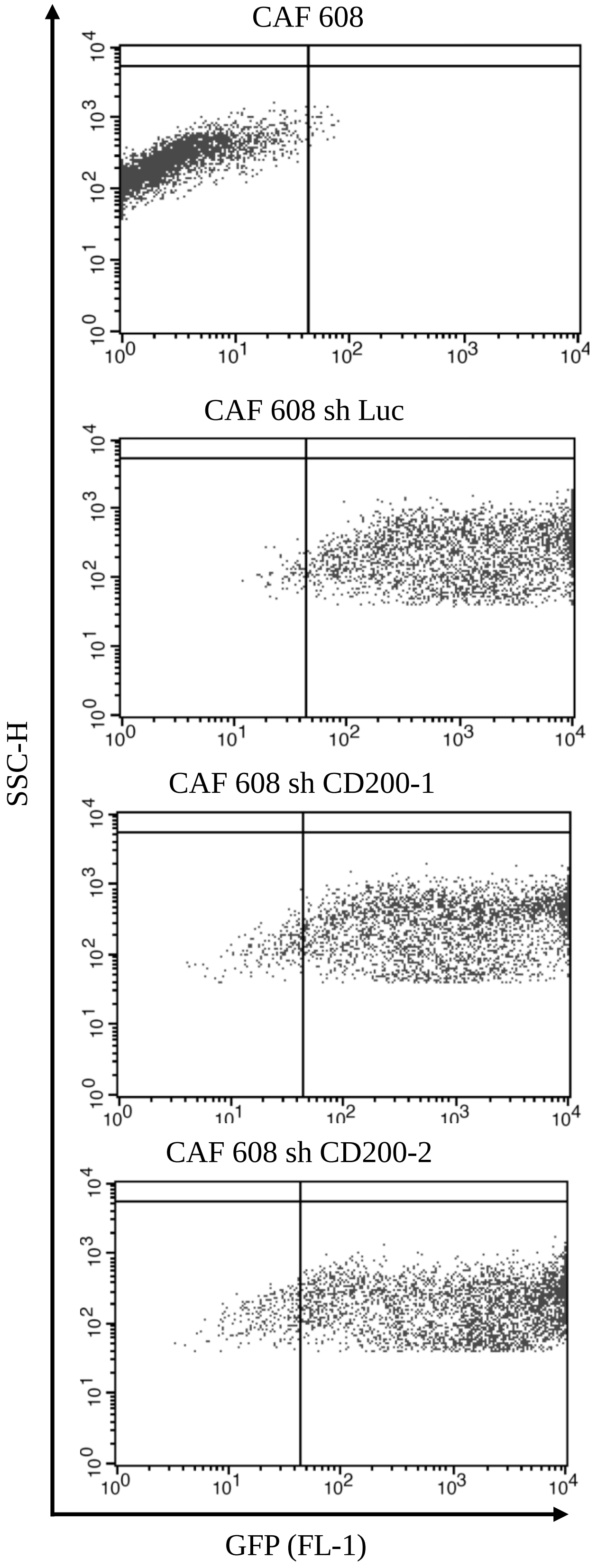

Supplemental Figure 7

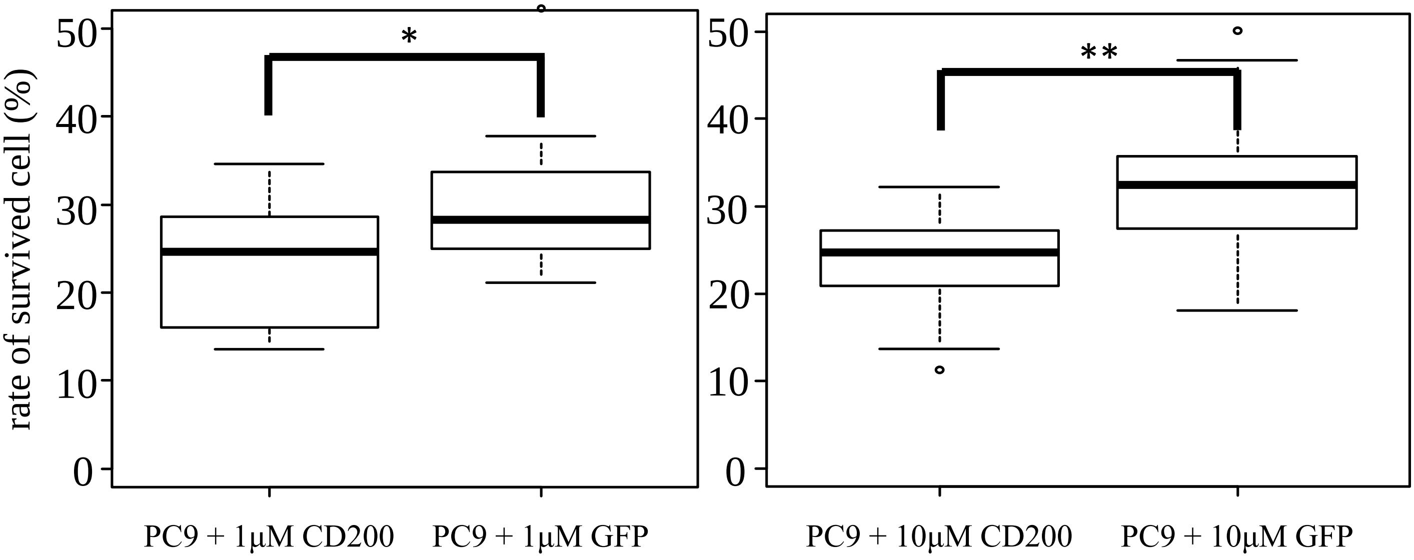

**Supplemental table 1. The clinicopathological informations.**

| sample | age | gender | histopathology | stage | EGFR gene mutation in carcinoma cell | histological feature |
|---|---|---|---|---|---|---|
| CAF608 | 65 | male | Adenocarcinoma | IIIB | wild | micropapillary |
| CAF722 | 68 | male | Adenocarcinoma | IIIA | wild | solid with mucin |
| CAF1128 | 69 | male | Adenocarcinoma | IIIB | wild | acinar |
| CAF621 | 61 | male | Adenocarcinoma | IIIA | L858R | solid with mucin |
| CAF1209 | 64 | male | Adenocarcinoma | IIB | exon19del | papillary |

Supplemental Table 2 The sequences of primer RT-PCR.

| Primer | | | | |
|---|---|---|---|---|
| HGF | Forward | 5’- | CTCACACCCGCTGGGAGTAC | -3’ |
| | Reverse | 5’- | TCCTTGACCTTGGATGCATTC | -3’ |
| CD200 | Forward | 5’- | GTTTGGGTCATGGCAGCAGT | -3’ |
| | Reverse | 5’- | CCATGTCACAATGAGGGCTTC | -3’ |
| NRCAM | Forward | 5’- | CCTCACTGTCCTGTGGCTGAA | -3’ |
| | Reverse | 5’- | TAGGTCCCGCTGTCATCGTC | -3’ |
| GAPDH | Forward | 5’- | GCACCGTCAAGGCTGAGAAC | -3’ |
| | Reverse | 5’- | ATGGTGGTGAAGACGCCAGT | -3’ |

Supplemental Table 3  Lentiviral vectors used.

| LV vector | shRNA target gene | shRNA target sequence |
| --- | --- | --- |
| sh CD200-1 | CD200 | GGTGTTACATGTGTCTCTTCA |
| sh CD200-2 | CD200 | GCATTGTTTCCCTGGTAATTC |

Supplemental Table 4. Patient characteristics (n=90)

| | CD200 expression of CAFs | | |
|---|---|---|---|
| | Positive N (%) | Negative N (%) | p |
| Number of patients | 30 | 60 | |
| Age, y, median (range) | 63 (49-81) | 69 (42-85) | |
| Gender | | | |
| Male | 12 (40) | 26 (43) | 0.823 |
| Female | 18 (60) | 34 (57) | |
| Performance status | | | |
| 0/1 | 29 (97) | 53 (88) | 0.261 |
| 2/3 | 1 (3) | 7 (12) | |
| Smoking status | | | |
| Never smoker | 19 (63) | 35 (58) | 0.820 |
| Current+former smoker | 11 (37) | 25 (42) | |
| Smoking index | | | |
| Median (range) | 0 (0-930) | 0 (0-2880) | |
| Adjuvant chemotherapy | | | |
| yes/no | 9 (30) / 21 (70) | 22 (37) /38 (63) | 0.640 |
| EGFR mutation | | | |
| Exon 19 deletion | 16 (54) | 24 (40) | 0.265 |
| L858R | 13 (43) | 34 (58) | |
| L861Q | 0 | 1 (1) | |
| G719C | 1 (3) | 1 (1) | |
